# Case Report: Successful Treatment of Five Critically Ill Coronavirus Disease 2019 Patients Using Combination Therapy With Etoposide and Corticosteroids

**DOI:** 10.3389/fmed.2021.718641

**Published:** 2021-09-23

**Authors:** Tetsuji Aoyagi, Yukio Sato, Hiroaki Baba, Takuya Shiga, Issei Seike, Ikumi Niitsuma Sugaya, Kentarou Takei, Yudai Iwasaki, Kengo Oshima, Hajime Kanamori, Makiko Yoshida, Koji Saito, Koichi Tokuda, Mitsuo Kaku

**Affiliations:** ^1^Department of Infectious Diseases, Internal Medicine, Tohoku University Graduate School of Medicine, Sendai, Japan; ^2^Department of Intelligent Network for Infection Control, Tohoku University Graduate School of Medicine, Sendai, Japan; ^3^Department of Intensive Care Unit, Tohoku University Hospital, Sendai, Japan; ^4^Department of Anaesthesiology and Perioperative Medicine, Tohoku University Graduate School of Medicine, Sendai, Japan; ^5^Division of Infectious Diseases and Infection Control, Tohoku Medical and Pharmaceutical University, Sendai, Japan

**Keywords:** COVID-19, SARS-CoV-2, etoposide, corticosteroid, case series, acute respiratory distress (ARDS)

## Abstract

Acute respiratory distress syndrome (ARDS) is the leading cause of mortality in hospitalized patients with coronavirus disease 2019 (COVID-19) because of limited effective therapies. During infection, the accumulation and activation of macrophages and monocytes in the lungs induce inflammatory mediators and contribute to tissue injury, leading to ARDS. However, therapeutic strategies that directly target activated macrophage and monocytes have not been reported. Combination treatment with etoposide (a cytotoxic agent) and a corticosteroid has been widely used for treating hemophagocytic lymphohistiocytosis characterized by the systemic activation of macrophages with overwhelming inflammation. Herein, we present five cases of COVID-19-associated ARDS treated with etoposide and corticosteroids. Three of the five patients were over 65 years of age and had various underlying diseases, including multiple myeloma. Four patients required invasive mechanical ventilation (MV), and one patient refused to be placed on MV due to underlying diseases. All patients were pre-treated with antiviral and/or other anti-inflammatory agents, but their condition deteriorated and hyperinflammation was noted. All five patients responded well to treatment and had an immediate response, as reflected by improvement in their respiratory condition and inflammatory marker levels and rapid resolution of fever after etoposide administration; however, some patients required a second dose of etoposide and longer course of steroids. All patients recovered, and there were no severe adverse events related to the drugs. Following successful treatment in these five patients, we plan to conduct a clinical trial to evaluate the efficacy and safety of combination therapy with etoposide and corticosteroid for treating COVID-19 patients in Japan.

## Introduction

Acute respiratory distress syndrome (ARDS) is the leading cause of mortality among critically ill coronavirus disease 2019 (COVID-19) patients (1). Disease severity or mortality in COVID-19 patients is associated with elevated levels of pro-inflammatory cytokines and chemokines. Corticosteroid therapy is widely used to suppress inflammatory mediators in COVID-19 and has been reported to improve survival, especially in critically ill patients requiring invasive mechanical ventilation (MV) (2, 3). However, the associated mortality rate remains as high as 30% (2, 3).

Hemophagocytic lymphohistiocytosis (HLH) is a potentially life-threatening hyperinflammatory syndrome characterized by sustained activation of macrophages and elevated cytokine levels. Histologic evidence of secondary HLH (sHLH) has been observed in fatal respiratory infection with severe acute respiratory syndrome coronavirus 1, H5N1, and 2009H1N1pdm influenza virus (4–6). Recently, post-mortem analysis of COVID-19 patients with ARDS showed diffuse alveolar damage and hemophagocytosis with pulmonary hilar or mediastinal lymphadenopathy (7). Clinical data of severe COVID-19 patients revealed lymphopenia, hyperferritinemia, and elevated pro-inflammatory cytokine levels (8). Thus, macrophage-mediated inflammation might be involved in the pathogenesis of ARDS caused by severe acute respiratory syndrome coronavirus 2 (SARS-CoV-2) infection.

Historically, the HLH-94 protocol, etoposide (a cytotoxic agent), and a corticosteroid have been used to treat primary or secondary HLH (9). Etoposide is a chemotherapeutic drug that inhibits topoisomerase II, resulting in DNA synthesis errors that lead to apoptosis in rapidly dividing or activated cells. In this process of etoposide-induced cell death, etoposide can reduce the number of macrophages and monocytes in blood. We previously demonstrated that a combination therapy using etoposide and a low-dose corticosteroid, but not etoposide or a corticosteroid alone, reduced the mortality rate and lung injury in a fatal ARDS murine model, in which hypercytokinemia and highly activated macrophages with hemophagocytic activity were observed (10). Moreover, successful treatment with a combination of etoposide and corticosteroid has been reported in severe ARDS caused by H1N1/09 influenza virus (11). Recently, in a study of 11 patients with COVID-19 without invasive MV treated with etoposide as salvage therapy (12), all patients received methylprednisolone plus Tocilizmab (IL-6 inhibitor) or Ankinra (IL-1 inhibitor) before treatment with etoposide. Herein, we report the disease course of five critically ill COVID-19 patients on invasive MV who were treated with a combination of etoposide and corticosteroids.

## Case Description

**Figure 1:**
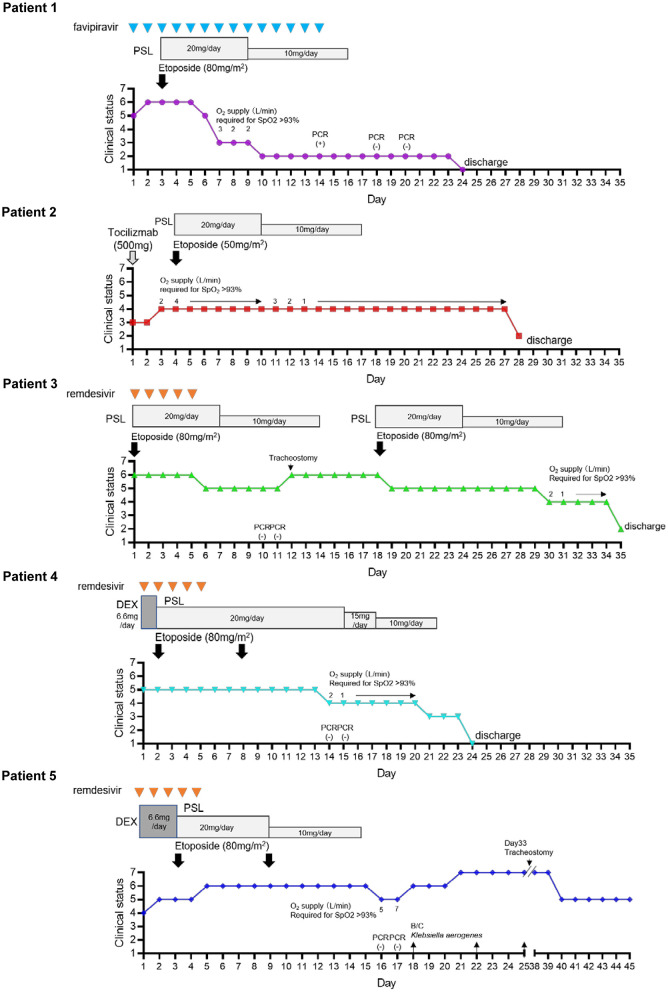
Timeline of clinical progress of COVID-19 patients after hospitalization and that after treatment with etoposide and corticosteroid. An 7 point ordinal clinical scale was used to assess pulmonary status consisting of the following values: 7, Ventilation in addition to extracorporeal membrane oxygen (ECMO); 6, Ventilation in addition to need for vasopressors (noradrenaline ≥ 0.1 μg/kg/min); 5, Intubation and mechanical ventilation; 4, Oxygen by mask or nasal prongs; 3, Hospitalization without oxygen supplementation; 2, Discharged from hospital either to home or to inpatient rehabilitation facility with supplemental oxygen; 1, Discharged to home without supplemental oxygen.

**Figure 2:**
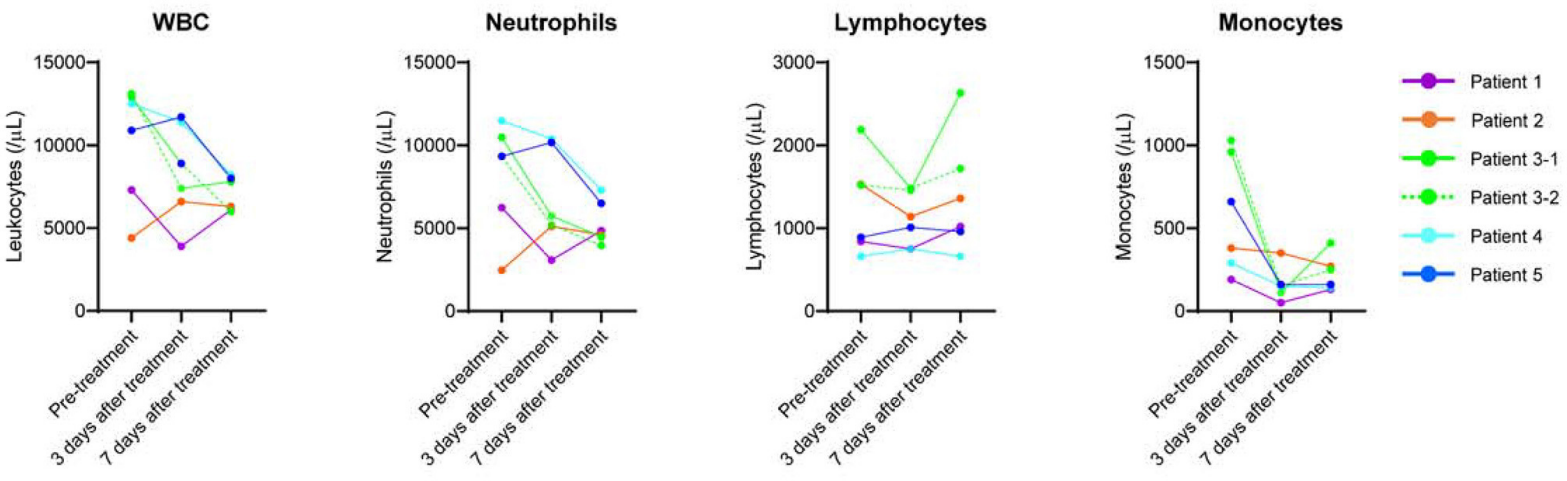
Changes in white blood cell count before and after treatment with etoposide and corticosteroid.

The off-label use of etoposide and corticosteroid was approved by the Ethics Committee of Tohoku University Hospital. All patients received heparin (10,000–20,000 U/day) to prevent thrombosis. In patients undergoing invasive MV, prone positioning was performed. Figure 1; Table 1 show the clinical progress and clinical characteristics of five critically ill patients infected with SARS-CoV-2, respectively. We evaluated clinical indicators for activation of macrophages and monocytes using H-Score and COVID-19-associated hyperinflammatory syndrome (cHIS) score (Table 1). The median value of H-Score, excluding histological findings of bone marrow, was 54 (range: 33–97) on administration of etoposide and corticosteroid. All patients met 2–4 items of the cHIS criteria at the time of etoposide administration. The median sequential organ failure assessment (SOFA) score at the time of etoposide administration was 5.5 (range: 3–8), which improved by 0.5 (0–2) after a combination treatment with etoposide and prednisolone (PSL). Figure 2 shows the changes in white blood cell count before and after treatment with etoposide and corticosteroid.

**Table 1:** Patient background, laboratory parameter, time-course of admission, and assessment of clinical outcome after etoposide and corticosteroid.

	**Patient 1**	**Patient 2**	**Patient 3**		**Patient 4**	**Patient 5**
Age	58	88	71		45	69
Co-morbidity	None	Multiple myeloma, CKD, DM, HT	Brain infarction, hemiplegia, atrial fibrillation, CHF, HT, DM		Childhood asthma	Post-CABG, stent graft for abdominal aortic aneurysm, brain infarction, HL, HT, DM
Current or Ex-smoker	YES	NO	YES		NO	YES
Sex	Female	Female	Male		Male	Male
Fever (>38°C)	YES	YES	YES^*^	YES^**^	YES	YES
PaO_2_/FiO_2_ (mmHg)	134	250	90	183	150	104
Ferritin (ng/ml)	1,507	538.6	323.6	855	588.1	580.3
soluble IL-2R (U/ml)	630	954	1262	1205	883	1,453
CRP (mg/dl)	3.91	3.14	15.93	20.88	19.16	10.46
LDH (U/L)	509	349	306	317	288	193
D-dimer (μg/ml)	1.7	3.1	1.3	2.8	1.1	4.7
CK (U/L)	797	55	58	62	27	144
Neutrophils (/μl)	6,240	2,470	10,490	9,340	10,390	13,450
Lymphocytes (/μl)	620	1,530	1,520	1,480	660	760
Cytopenia > 2 Lines	NO	NO	NO	NO	NO	NO
Hepatomegaly or Splenomegaly	NO	NO	NO	NO	Splenomegaly	NO
H-Score (without histological evidence of hemophagocytosis)	96	97	33	52	56	33
cHIS score	4/6	3/6	2/6	3/6	3/6	3/6
Mechanical ventilation	YES	NO	YES	YES	YES	YES (ECMO)
Etoposide (doses and times)	80 mg/m^2^ × 1	50 mg/m^2^ × 1	80 mg/m^2^ × 1	80 mg/m^2^ × 1	80 mg/m^2^ × 2	80 mg/m^2^ × 2
Prednisolone (dose and duration)	20 mg for 1 week → 10 mg for 1 week	20 mg for 1 week → 10 mg for 1 week	20 mg for 1 week → 10 mg for 1 week	20 mg for 1 week → 10 mg for 1 week	20 mg for 2 week → 15 mg for 3 days → 10 mg for 6 days	20 mg for 1 week → 10 mg for 1 week
Post etoposide and prednisolone PaO_2_/FiO_2_ (mmHg)	480	450	396	397	450	250
A change in SOFA score (before and after etoposide and prednisolone)	7 to 0	4 to 0	8 to 1	7 to 1	3 to 0	4 to 2
Outcome	Discharge	Discharge	Discharge		Discharge	Continue rehabilitation
Infection	NO	NO	NO	NO	NO	YES (perianal abscess)

* *First course*,

** *Second course*.

*H-Score; (20)*.

*cHIS; (24)*.

*CKD, chronic kidney disease; DM, diabetes mellitus; HT, hypertension; CHF, chronic heart failure; HL, hyperlipidemia; CABG, Coronary artery bypass grafting; ECMO, extracorporeal membrane oxygenation; SOFA, sequential organ failure assessment*.

### Patient 1

In April 2020, a 58-year-old woman was transferred to our hospital with severe hypoxia (SpO_2_ 90% on 8 L/min oxygen) 7 days after the onset of COVID-19 symptoms, which included general fatigue and cough. She was immediately transferred to our intensive care unit (ICU) and underwent endotracheal intubation and MV. Chest computed tomography (CT) showed bilateral ground-glass opacities and peripheral condensation. Partial pressure of oxygen in arterial blood (PaO_2_) was 80 mmHg while receiving FiO_2_ of 60%, which yielded a PaO_2_ to FiO_2_ ratio (P/F ratio) of 134 mmHg. She was treated with favipiravir (3,600 mg on day 1, followed by 1,800 mg from days 2 to 14). On day 2, noradrenaline was administered to maintain a mean blood pressure of >70 mmHg. On day 3, she still had fever (>38°C); her general condition deteriorated, with decreased urine output, and an increased dose of noradrenaline was required. Moreover, her laboratory data showed increased levels of soluble interleukin-2 receptor (sIL-2R), C-reactive protein (CRP), and creatine kinase (CK). We decided to treat her with etoposide (80 mg/m^2^) and PSL (20 mg/day) (instead of dexamethasone as PSL is the preferred corticosteroid in Japan). On the day following treatment with etoposide and corticosteroids, her respiratory and hemodynamic conditions improved dramatically, and the P/F ratio (312 mmHg) increased significantly. On day 7, 4 days after starting treatment, she was extubated. Because her general condition continued to be good and laboratory data showed decreased inflammatory marker levels, including sIL-2R and CRP, the PSL dose was tapered after 7 days of treatment. The patient was discharged home without supplemental oxygen after real-time polymerase chain reaction (RT-PCR) tests for SARS-CoV-2 on days 18 and 20 were confirmed to be negative.

### Patient 2

An 88-year-old woman with a history of recently diagnosed multiple myeloma (Bence-Jones protein λ, R-ISS III), diabetes mellitus, and chronic kidney failure (serum creatinine, 2.15 mg/dl) presented with low-grade fever and exertional dyspnea owing to COVID-19 pneumonia, with SpO_2_ of 95% on room air at presentation. Chest CT revealed bilateral multiple ground-glass opacities. Her renal function prevented the use of antiviral agents, including remdesivir. A single dose of 500 mg tocilizumab was administered on the day of admission. However, on day 4 of hospitalization, she developed fever (>38°C), and her respiratory condition progressively worsened (P/F ratio: 252 mmHg), but her family did not agree to MV. The patient was treated with etoposide and PSL (20 mg/day). The dose of etoposide was reduced from 80 to 50 mg/m^2^, considering her kidney failure and old age. After this treatment, her fever subsided and her respiratory condition gradually improved (P/F ratio: 452 mmHg). However, she still had exertional dyspnea, and CT showed progressive consolidation and parenchymal changes with pulmonary fibrosis. On day 28, she was discharged home with supplemental oxygen therapy. She was followed up in the outpatient clinic for 3 months with no evidence of exacerbation. However, she could not be weaned from home oxygen therapy.

### Patient 3

A 71-year-old man with a history of hemiplegia owing to a stroke, atrial fibrillation, hypertension, chronic heart failure, and untreated diabetes mellitus was intubated and transferred to our hospital because of severe hypoxia and fever (>38°C) at 10 days after the laboratory diagnosis of asymptomatic SARS-CoV-2 infection following close contact with another patient. CT showed lung consolidation with bronchial distribution in multiple lobes and segments and thrombus formation in the left atrial appendage. He was immediately admitted to our ICU and required invasive MV for severe ARDS with a P/F ratio of 90 mmHg; noradrenalin was administered to maintain his blood pressure. Treatment with remdesivir (200 mg loading dose on day 1, followed by 100 mg daily for up to 4 additional days), etoposide (80 mg/m^2^), and PSL (20 mg/day) was started. On the day after etoposide administration, his fever subsided, respiratory and hemodynamic conditions improved dramatically, and P/F ratio increased to 152 mmHg. Noradrenalin was discontinued 5 days after treatment initiation. His general condition and laboratory data, including lactate dehydrogenase, CK, sIL-2R, and CRP levels, remarkably improved. The PSL dose was tapered after 7 days of treatment. RT-PCR tests for SARS-CoV-2 on days 10 and 11 were confirmed to be negative. However, on day 12, he developed high fever (>39°C), and circulatory agents, including noradrenalin and dobutamine, were required to maintain his hemodynamic condition because he had a history of atrial fibrillation and chronic heart failure; tracheostomy was performed. There was no evidence of superinfection. Several blood and sputum cultures were negative, and CT showed improved lung consolidation with no new infectious sites. There were clinical and biochemical features of cytokine-releasing syndrome, with increased levels of aspartate transaminase, lactate dehydrogenase, sIL-2R, ferritin, D-dimer, and CRP levels and increased peripheral monocytes. Thus, a second course of etoposide and PSL was administered for macrophage activation syndrome on day 18. The day after starting the second treatment course, his fever subsided and general and hemodynamic conditions improved immediately; the PSL dose was tapered 7 days after second course of etoposide. He was weaned from the MV 10 days post-second treatment course (on day 29) and discharged to an inpatient rehabilitation hospital with supplemental oxygen therapy on day 35. Four months later, he was discharged home without oxygen support.

### Patient 4

A 45-year-old man with a history of childhood asthma presented with fever (>39°C), cough, and dyspnea 10 days after the onset of symptoms. His SpO_2_ was 90% on 10 L/min oxygen *via* a mask. Chest CT showed consolidation with a bronchial distribution in multiple lobes. SARS-CoV-2 infection was confirmed on a respiratory viral multiplex PCR test using a nasal swab sample. Dexamethasone (6.6 mg/day) and favipiravir were started immediately after diagnosis. On the day after treatment initiation, his respiratory condition deteriorated owing to ARDS and fever remained high (>39°C). He was transferred to our hospital for further management. He was intubated and admitted to the ICU, and favipiravir was replaced with remdesivir. On day 2, his respiratory condition had not improved (P/F ratio: 150 mmHg) and high fever persisted; thus, etoposide (80 mg/m^2^) and PSL (20 mg/day) were administered. His P/F ratio improved to 220 mmHg and fever subsided 2 days after combination treatment initiation. On day 6, he developed high fever (>39°C), and his P/F ratio worsened to 152 mmHg with no evidence of superinfection (negative blood and sputum cultures). On day 9, CT revealed worsening pulmonary consolidation, mediastinal lymphadenopathy, and splenomegaly. Laboratory test results showed increased sIL-2R (1506 U/ml), ferritin (991.3 ng/ml), CRP (24.4 mg/dl), and D-dimer (2.9 g/ml) levels in contrast to the levels before combination treatment initiation. Thus, we decided to administer a second dose of etoposide and continue the same dose of PSL for another week. His fever eventually subsided and P/F ratio improved to 220 mmHg 2 days after the second dose of etoposide; he was extubated on day 13. RT-PCR tests for SARS-CoV-2 on days 14 and 15 were confirmed to be negative. Because his general condition continued to be good and laboratory data showed decreased inflammatory marker levels, the PSL dose was gradually tapered after 14 days of treatment and stopped on day 20.

He was discharged home without supplemental oxygen on day 24.

### Patient 5

A 68-year-old man with a history of coronary artery bypass surgery, endovascular aortic repair for an abdominal aortic aneurysm, stroke, diabetes mellitus, hypertension, and hyperlipidemia presented with general fatigue and dyspnea 5 days after the onset of symptoms including low-grade fever, cough, and rhinorrhea. His SpO_2_ was 94% on 1 L/min oxygen via a nasal tube. Chest CT showed bilateral multiple ground-glass opacities. Laboratory data showed an elevated D-dimer level (4.7 g/ml). Treatment with dexamethasone (6.6 mg/day), favipiravir, and heparin (10,000 U/day) was immediately started after SARS-CoV-2 infection was confirmed by respiratory viral multiplex PCR. However, his respiratory condition (SpO_2_ 90–95% on 5 L/min oxygen *via* a mask) deteriorated 3 days after treatment initiation. He was transferred to our hospital for further management, and favipiravir was replaced with remdesivir. On day 2, he developed high fever (>39°C) and his SpO_2_ decreased to <90% on 10 L/min oxygen *via* a mask. Chest CT revealed progression of the pulmonary lesions in extent and density. He was transferred to our ICU and required invasive MV for severe ARDS with a P/F ratio of 105 mmHg. On day 3, his respiratory condition and laboratory data, including inflammatory marker levels, had not improved despite dexamethasone was being administered for 5 days; hence, etoposide (80 mg/m^2^) and PSL (20 mg/day) were administered. His P/F ratio improved to 237.5 mmHg and fever subsided 2 days after combination therapy initiation. On day 10, he developed high fever again, and laboratory data showed increased inflammatory marker levels, including sIL-2R (1741 U/ml), CRP (11.39 mg/dl), and D-dimer (5.1 g/ml), but no evidence of secondary infection or change in respiratory condition was noted. Thus, we administered a second dose of etoposide and tapered the PSL dose to 10 mg. After the second dose of etoposide, his fever subsided immediately; he was extubated on day 15. SARS-CoV-2 PCR tests were confirmed to be negative on days 16 and 17. On day 18, he developed septic shock and was re-intubated, and noradrenaline was administered. Blood and central catheter line samples were positive for *Enterobacter aerogenes*. On day 21, his respiratory condition worsened (P/F ratio: 100 mmHg) even though carbapenem and an aminoglycoside were administered, necessitating venovenous extracorporeal membrane oxygenation (ECMO). *E. aerogenes* was isolated several times from the blood culture, despite effective antimicrobial therapy and catheter replacement. Eventually, CT showed a perianal abscess. Drainage of the abscess and administration of antimicrobial agents improved his condition, and he was weaned off ECMO on day 40. Three months later, he was weaned off the mechanical ventilator and transferred to an inpatient rehabilitation hospital.

## Discussion

Expanding the treatment options for critically ill COVID-19 patients on MV will improve individual outcomes and reduce the burden on medical practitioners and healthcare staff leading to more effective use of medical resources. The COVID-19 mortality rate is particularly high in elderly patients requiring invasive MV (13). Combination treatment with etoposide and low-dose corticosteroid showed favorable outcomes in five cases of severe COVID-19, including four patients on invasive MV. Three of the five patients who received etoposide and corticosteroid were over 65 years of age and had various underlying diseases, but all of them recovered. The 88-year-old patient with multiple myeloma was not placed on invasive MV during treatment.

Peripheral blood in patients with severe COVID-19 shows lymphopenia with increased CD14-positive monocyte and inflammatory cytokine levels, such as tumor necrosis factor (TNF)-α and interferon (IFN)-γ (14, 15). In severe COVID-19 pneumonia, TNF-α and IFN-γ induce nitric oxide synthase (*i*NOS), which produces cytotoxic nitric oxide in macrophages and plays a central role in tissue injury and mortality (16). Thus, macrophages and monocytes are implicated as potential therapeutic targets to reduce hyperinflammation and tissue injury. Using a lethal ARDS model, we demonstrated that combination therapy with etoposide and low-dose corticosteroids improved mortality and lung injury associated with suppressed macrophage/monocyte accumulation and high *i*NOS expression levels in the lungs rather than suppressed inflammatory cytokines (10). In our COVID-19 cases, treatment with etoposide and corticosteroid rapidly reduced the number of circulating monocytes (Figure 2) and improved the respiratory condition.

Etoposide is an anti-cancer agent; thus, clinicians should be aware of its dose-dependent side effects. When the HLH-94 protocol is applied to adults with HLH, reducing the etoposide dose from 150 mg/m^2^ to 50–100 mg/m^2^ is recommended (9). Thus, we administered etoposide at a dose of 80 mg/m^2^ in all patients, except the one with impaired renal function. Secondary leukemia is one of the most serious side effects of etoposide, occurring in up to 18.4% of patients treated with cumulative doses ranging from 5,200 to 19,200 mg/m^2^ (17). We treated five COVID-19 patients with 50–80 mg/m^2^ of etoposide as one or two doses. This dose is much lower than the treatment dose for small lung cancer and that in the HLH-94 protocol. Reactive cytopenia and hepatotoxicity are other potential adverse events following etoposide administration. However, these potential side effects were not observed in any of our patients. We believe that etoposide can be used safely to treat patients with severe COVID-19 who are on invasive MV; however, a clinical trial is needed to validate the efficacy and safety of this regimen.

Dexamethasone is effective in severe COVID-19. The recommended dose of dexamethasone (6 mg/day) is equivalent to 40 mg/day of PSL. High-dose corticosteroid (>20 mg/day PSL) increases the risk of steroid toxicity, including diabetes mellitus and secondary infection (18). Further, the administration of high-dose corticosteroids to COVID-19 patients increases the risk of secondary infections, including bacteremia and pulmonary aspergillosis (3, 19). Therefore, we decided to administer low-dose corticosteroids (20 mg/day for 1 week, subsequently reduced to 10 mg/day for 1 week) based on animal experiment findings (10). One patient had perianal abscess-associated bacteremia, but the corticosteroid dose could be reduced by combining it with etoposide. Moreover, corticosteroids affect glycemic control in individuals with diabetes or hyperglycemia due to COVID-19 (3, 19). Three patients had underlying diabetes mellitus, and their glucose levels increased after combination treatment with etoposide and corticosteroids that could be controlled with insulin administration. The other two patients did not show abnormal glucoses levels during the therapy.

Previous studies demonstrated that ICU admission and corticosteroid treatment significantly delayed viral clearance (20, 21). All the four ICU patients had negative SARS-CoV-2 RT-PCR tests on days 10–18 after hospital admission (Figure 1). Among COVID-19 ICU patients, the median time to a negative SARS-CoV-2 RT-PCR test was 19–26 days post-ICU admission (22). Thus, it is unlikely that our combination therapy delayed viral clearance when compared with other immunosuppressive therapies.

The H-Score has been widely used to support the diagnosis of sHLH (23). However, none of our patients met the sHLH criterion using the H-Score upon etoposide and corticosteroid administration (Table 1). In a study of autopsy cases of H1N1/09 influenza, <50% with histological evidence of hemophagocytosis met the criteria for sHLH based on the H-S score (6). Moreover, the H-Score was low in COVID-19 patients with hemophagocytosis (7). These discrepancies may be explained by the fact that the lung is the initial site of hyperinflammation following respiratory virus infection, thus HLH occurs in the lung, but not always throughout the body as is observed in malignancies and autoimmune diseases. Recently, Webb et al. proposed a set of six clinical criteria for cHIS: fever, macrophage activation (hyperferritinemia), hematological dysfunction (neutrophil-to-lymphocyte ratio), hepatic injury (lactate dehydrogenase or aspartate aminotransferase), coagulopathy (D-dimer), and cytokinemia (C-reactive protein, interleukin-6, or triglycerides). Meeting two or more of the six cHIS items is generally associated with severe disease and high fatality rate (24). All our patients met more than two of the cHIS items at the time of etoposide administration. Although these criteria need to be validated, they may serve as an indicator for clinicians to consider life-saving immunotherapy, including etoposide and corticosteroid, in severe COVID-19.

In conclusion, in this preliminary experience, combination treatment with etoposide and low-dose corticosteroids resulted in an overall favorable outcome in patients with SARS-CoV-2 infection-associated ARDS. Recently, etoposide has been described as an effective treatment for severe COVID-19 patients who do not require MV (12, 25). We plan to conduct a clinical trial to evaluate the efficacy and safety of combination therapy with etoposide and low-dose corticosteroids in critically ill COVID-19 patients who are on invasive MV in Japan (jRCT2021210012).

## Data Availability Statement

The original contributions presented in the study are included in the article/Supplementary Material, further inquiries can be directed to the corresponding author/s.

## Ethics Statement

The off-label use of etoposide and corticosteroid was approved by the Ethics Committee of Tohoku University Hospital. Written informed consent was obtained from patients. If the patients with ARDS received mechanical ventilation under the sedation, and they were not able to make their decision by themselves, we obtained written informed consent from their legal proxy for medical decision making before treatment. Written informed consent was obtained from the individual(s) for publication of any potentially identifiable images or data included in this articles.

## Author Contributions

TA, YS, and MK designed the study. TA and YS wrote the initial draft. HB, TS, IS, IN, KTa, YI, KO, HK, MY, KS, and KTo provided patient care and collected the data. This paper has been reviewed, edited, and approved by all authors.

## Funding

This study has been supported in part by Japanese Society for the Promotion of Science Grant-in-Aid for Scientific Research 18K08424 (TA). Department of Intelligent Network for Infection Control, Tohoku University Graduate School of Medicine (TA, HB, KO, HK, MY, KTo) is an endowment department, supported with an unrestricted grant from Kyosei Igaku Laboratory Co., Ltd. The funder was not involved in the study design, collection, analysis, interpretation of data, the writing of this article or the decision to submit it for publication.

## Conflict of Interest

The authors declare that the research was conducted in the absence of any commercial or financial relationships that could be construed as a potential conflict of interest.

## Publisher's Note

All claims expressed in this article are solely those of the authors and do not necessarily represent those of their affiliated organizations, or those of the publisher, the editors and the reviewers. Any product that may be evaluated in this article, or claim that may be made by its manufacturer, is not guaranteed or endorsed by the publisher.
